# Management of hypertensive crisis in a patient with underlying kidney disease: A case report

**DOI:** 10.1097/MD.0000000000036152

**Published:** 2023-11-17

**Authors:** Chukwuka Elendu, Dependable C. Amaechi, Tochi C. Elendu, Yuliana Paola Oros Sucari, Sehajmeet Kaur Saggi, Kanishk Dang, Jennifer O. Ibhiedu

**Affiliations:** a University of California, Santa Cruz, United States; b Igbinedion University Okada, Benin City, Nigeria; c Imo State University, Owerri, Nigeria; d Universidad peruana Cayetano Heredia, San Martín de Porres, Peru; e Belarusian State Medical University, Minsk, Belarus; f Nicolae Testemițanu State University of Medicine and Pharmacy, Chişinău, Republic of Moldova; g Babcock University, Ilishan-Remo, Nigeria.

**Keywords:** case report, diagnoses, hypertensive crisis, interventions, management, underlying kidney disease

## Abstract

**Rationale:**

This case report elucidates the management of a hypertensive crisis in a patient with underlying kidney disease, shedding light on the intricate interplay between these conditions. This unique case contributes valuable insights to the scientific literature.

**Patient Concerns:**

The patient exhibited severe headache, visual disturbances, and chest pain. Clinical evaluation revealed elevated blood pressure and impaired kidney function, emphasizing the importance of monitoring hypertension and renal health in such cases.

**Diagnoses and Interventions:**

The primary diagnoses included malignant hypertension and underlying kidney disease. Immediate interventions comprised intravenous antihypertensive agents and rigorous hemodynamic monitoring, yielding favorable outcomes. Blood pressure gradually returned to acceptable levels, and renal function improved during treatment.

**Conclusions:**

This case underscores the critical need for timely recognition and management of hypertensive crises in patients with preexisting kidney dysfunction. Simultaneously addressing both conditions is vital for successful outcomes. Healthcare practitioners must remain vigilant in assessing the intricate relationship between hypertension and kidney disease, employing tailored interventions for optimal results.

**Lesson Learned:**

The primary lesson from this case is the necessity of a comprehensive approach to managing hypertensive crises in individuals with underlying kidney disease. Early intervention and a multidisciplinary strategy are essential to achieve positive clinical outcomes and prevent potential complications.

## 1. Introduction

This case report presents a unique and clinically significant scenario that delves into the complex interplay between hypertensive crises and underlying kidney disease. Hypertension and kidney dysfunction are inherently intertwined, often exacerbating the other and leading to potentially life-threatening complications. The distinctiveness of this case lies in its spotlight on the intricate relationship between these conditions, offering valuable insights that contribute to the evolving landscape of medical knowledge.

Hypertensive crises, characterized by a rapid and severe increase in blood pressure, can precipitate organ damage and dysfunction, particularly in patients with underlying kidney disease.^[1]^ In such cases, the challenge lies in managing acute blood pressure surges and addressing the potential exacerbation of renal impairment. While a body of literature discusses hypertensive crises and kidney disease separately, this case report bridges the gap by illustrating their symbiotic interaction and the need for tailored interventions.

This case report aims to contribute to the scientific community’s understanding of effective management strategies for hypertensive crises in patients with underlying kidney disease by presenting a comprehensive analysis of clinical findings, diagnoses, and therapeutic approaches. The insights from this case emphasize the importance of a multidisciplinary approach, integrating knowledge from cardiology, nephrology, and emergency medicine to optimize patient outcomes.

## 2. Patient information

The 52-year-old male patient presented to the emergency department with a history of underlying kidney disease and primary concerns of severe headache, visual disturbances, and chest pain. Given the patient’s medical history of hypertension and renal dysfunction, these symptoms prompted immediate medical attention. The patient’s genetic predisposition to hypertension, as indicated by a family history of cardiovascular diseases, added complexity to the diagnostic and management considerations.

### 2.1. Medical history

The patient’s medical history revealed a longstanding diagnosis of chronic kidney disease (Stage 3) attributed to hypertensive nephropathy. He had been under the care of a nephrologist for the past 5 years, receiving regular monitoring and conservative management. The patient’s hypertension had been managed with antihypertensive medications, primarily angiotensin-converting enzyme inhibitors (ACE inhibitors) and diuretics. However, periodic fluctuations in blood pressure levels have been noted, reflecting the challenging nature of controlling hypertension in the context of kidney disease.

### 2.2. Family and psycho-social history

Family history highlighted a genetic predisposition to hypertension, with both parents having a history of high blood pressure and cardiovascular disorders. The patient’s psycho-social history indicated moderate stress levels attributed to his medical condition and the need for ongoing medical management.

### 2.3. Relevant past interventions and outcomes

The patient had experienced occasional episodes of elevated blood pressure, requiring adjustments to his antihypertensive medication regimen. Previous interventions included lifestyle modifications, dietary restrictions, and medication adjustments. These interventions yielded variable outcomes, with some instances of successful blood pressure control and others necessitating prompt medical attention due to hypertensive crises. The patient’s renal function had shown periods of stability, but periodic decline had been observed, emphasizing the challenges of managing kidney function in hypertension.

In this case, the patient’s complex medical history, genetic predisposition, and challenges in managing hypertension and kidney disease underscore the intricate relationship between these conditions and the need for tailored interventions to mitigate potential complications.

## 3. Clinical findings

Upon physical examination (PE), several significant findings shed light on the patient’s condition. The patient appeared visibly distressed, displaying signs of discomfort and agitation. Blood pressure measurements revealed severely elevated levels, well beyond the normal range. The patient’s systolic blood pressure was measured at 200 mm Hg and diastolic blood pressure at 120 mm Hg, indicating a hypertensive crisis.

The patient’s cardiovascular examination revealed an irregular heart rate with a rapid pulse, suggesting the body’s compensatory response to the elevated blood pressure. Auscultation of the heart detected a systolic murmur, potentially indicative of underlying cardiac involvement. This murmur prompted further investigation into potential cardiac complications resulting from the hypertensive crisis.

Furthermore, an ophthalmoscopic examination of the patient’s retinas revealed hypertensive retinopathy characterized by arteriolar narrowing, flame-shaped hemorrhages, and cotton-wool spots. These retinal changes were consistent with the severity of the patient’s hypertensive crisis and highlighted the potential for end-organ damage.

The patient’s kidney function was assessed through laboratory tests, revealing elevated serum creatinine levels and reduced glomerular filtration rate (GFR), indicating worsening kidney function during the hypertensive crisis.

The PE findings collectively demonstrated the acute and severe nature of the hypertensive crisis, emphasizing the need for immediate therapeutic interventions to lower blood pressure and mitigate potential complications affecting the cardiovascular system, the eyes, and the kidneys.

## 4. Timeline of the episode of care

### 4.1. Preadmission period

The patient’s chronic kidney disease (Stage 3) and hypertension history are documented.

Previous episodes of blood pressure fluctuations were managed with medication adjustments.

### 4.2. Admission to the emergency department

The patient presents with severe headaches, visual disturbances, and chest pain.

Blood pressure was measured at 200/120 mm Hg, indicating a hypertensive crisis.

Cardiovascular examination reveals irregular heart rate and systolic murmur.

Ophthalmoscopic examination shows signs of hypertensive retinopathy.

Laboratory tests indicated elevated serum creatinine and reduced GFR.

### 4.3. Immediate management

Intravenous antihypertensive medications are administered to lower blood pressure rapidly.

Hemodynamic monitoring was initiated to assess the response to treatment.

Close monitoring of cardiac and renal function to prevent further complications.

### 4.4. Continued care

The patient stabilized over the next 24 hours, gradually decreasing blood pressure.

Regular monitoring of blood pressure, cardiac rhythm, and renal function.

Adjustment of antihypertensive medication regimen based on response and clinical status.

### 4.5. Cardiac evaluation

Cardiac imaging, including echocardiography, is performed to assess heart function.

Systolic murmur was investigated further to determine potential cardiac involvement.

No significant structural cardiac abnormalities were identified.

### 4.6. Ongoing nephrological assessment

Continued monitoring of kidney function through serial serum creatinine and GFR measurements.

Renal imaging to assess kidney perfusion and potential damage.

### 4.7. Resolution of hypertensive crisis

Blood pressure gradually stabilizes within acceptable ranges over several days.

Hypertensive retinopathy signs begin to improve with appropriate treatment.

### 4.8. Discharge and follow-up

The patient’s condition improves sufficiently for discharge.

Discharge instructions emphasize medication compliance and blood pressure monitoring.

Follow-up appointments are scheduled with a nephrologist and cardiologist to ensure ongoing management and prevent recurrence.

This timeline outlines the key events and interventions that transpired during the episode of care, illustrating the patient’s journey from presentation to stabilization and the subsequent plans for ongoing management and follow-up.

## 5. Diagnostic assessment

### 5.1. Diagnostic testing

*Physical examination (PE*): Assessment of vital signs, cardiovascular system, and retinal examination to identify signs of hypertensive crisis and end-organ damage.

*Laboratory testing*: Measure serum creatinine, GFR, electrolytes, and complete blood count to evaluate kidney function and potential metabolic derangements.

*Imaging*: Echocardiography to assess cardiac function and structure, renal imaging (ultrasound or CT) to evaluate kidney perfusion and identify anatomical abnormalities.

*Ophthalmoscopic examination*: To detect hypertensive retinopathy signs, such as arteriolar narrowing, hemorrhages, and cotton-wool spots.

### 5.2. Diagnostic challenges

*Access to testing*: Limited availability of immediate diagnostic tests in emergency settings.

*Financial constraints*: Potential financial burden associated with comprehensive diagnostic evaluations, especially imaging and specialized tests.

*Cultural factors*: Cultural beliefs and practices influence patient’s willingness to undergo certain diagnostic procedures.

### 5.3. Diagnosis

*Primary diagnosis*: Malignant hypertension with underlying chronic kidney disease.

*Other diagnoses considered*: Acute coronary syndrome, hypertensive emergency, and renal artery stenosis.

### 5.4. Prognosis

*Staging in oncology*: Not applicable in this case. However, the patient’s prognosis is assessed based on the severity of the hypertensive crisis, the extent of organ damage, and the response to immediate interventions.

*Renal function*: The prognosis involves monitoring the progression of kidney disease based on serial serum creatinine levels, GFR measurements, and response to treatment.

*Cardiovascular prognosis*: Ongoing assessment of cardiac function and potential complications to determine long-term cardiovascular outcomes.

The diagnostic assessment encompassed a range of tests and evaluations, including PE, laboratory tests, imaging, and ophthalmoscopic examination. Diagnostic challenges related to accessibility, financial considerations, and cultural factors may impact the comprehensive evaluation process. The primary diagnosis of malignant hypertension with underlying chronic kidney disease reflects the critical interplay between these conditions while considering other potential diagnoses ensures a thorough differential diagnosis approach. Prognosis is shaped by the severity of the hypertensive crisis and its impact on renal and cardiovascular health.

## 6. Therapeutic intervention

### 6.1. Pharmacologic interventions

*Intravenous antihypertensive medications*: Rapid-acting antihypertensive agents like nitroprusside or labetalol were administered to lower blood pressure acutely.

*Oral antihypertensive medications*: Following stabilization, the patient’s medication regimen was adjusted to include long-term management with ACE inhibitors, diuretics, and calcium channel blockers.

### 6.2. Preventive interventions

*Lifestyle modifications*: The patient received counseling on adopting a heart-healthy diet, low sodium intake, regular exercise, and stress reduction techniques to prevent future hypertensive crises.

*Medication adherence education*: The patient was educated about the importance of consistent medication use to manage hypertension and prevent recurrences.

### 6.3. Self-care interventions

*Home blood pressure monitoring*: The patient was instructed to monitor blood pressure regularly at home to ensure early detection of any fluctuations.

*Dietary modifications*: Recommendations for reduced sodium intake and balanced nutrition to support blood pressure management and kidney health.

### 6.4. Administration of therapeutic intervention

*Intravenous antihypertensive medications*: Nitroprusside infusion administered at 0.3 mcg/kg/min, titrated to reduce blood pressure over 24 hours gradually.

*Oral antihypertensive medications*: Dosages adjusted based on the patient’s response, including lisinopril 20 mg daily, hydrochlorothiazide 25 mg daily, and amlodipine 5 mg daily.

### 6.5. Changes in therapeutic intervention

*Medication adjustment*: The initial intravenous (IV) antihypertensive therapy was transitioned to oral agents as the patient’s condition stabilized, aiming for long-term blood pressure control.

*The rationale for change*: Transitioning to oral medications allows for sustained blood pressure management and minimizes the potential adverse effects of continuous IV therapy.

The therapeutic intervention encompassed both acute and long-term strategies to address the hypertensive crisis and manage underlying kidney disease. Pharmacologic interventions included IV and oral antihypertensive medications tailored to the patient’s response. Preventive measures emphasized lifestyle modifications and medication adherence to prevent recurrence. Self-care interventions empower the patient with tools to monitor blood pressure and make healthy choices. Changes in therapeutic interventions were made to transition from acute stabilization to long-term management, balance efficacy, and minimize potential risks.

## 7. Follow-up and outcomes

### 7.1. Clinician and patient-assessed outcomes

*Blood pressure control*: Blood pressure levels gradually normalized within target ranges over the course of hospitalization.

*Kidney function*: Serum creatinine levels and GFR showed improvement, indicating renal function stabilization.

### 7.2. Important follow-up diagnostic results

*Follow-up echocardiography*: Showed improved cardiac function without significant structural abnormalities.

*Renal imaging*: Confirmed adequate kidney perfusion and absence of acute damage.

### 7.3. Intervention adherence and tolerability

*Adherence assessment*: The patient’s adherence to the prescribed medication regimen was evaluated through patient interviews and pharmacy refill records.

*Tolerability assessment*: The patient’s response to medications was monitored for any adverse effects or complications.

### 7.4. Adverse and unanticipated events

*Hypotension episodes*: Following initial medication adjustments, the patient experienced transient episodes of hypotension, necessitating further dose modifications.

*Medication side effects*: The patient reported mild dizziness and dry cough as potential side effects of ACE inhibitor therapy.

### 7.5. Overall outcomes

*Blood pressure control*: The patient’s blood pressure remained within target ranges during hospitalization and at subsequent follow-up appointments.

*Kidney function*: Renal function improved, with serum creatinine levels returning to baseline and GFR stabilizing.

*Adherence and tolerability*: The patient demonstrated good adherence to the medication regimen, with only minor reported side effects.

The follow-up and outcomes demonstrated successful blood pressure management, improvement in renal function, and acceptable medication adherence. Clinician and patient-assessed outcomes collectively indicated positive progress in stabilizing the patient’s condition and preventing further complications. Follow-up diagnostic results confirmed the absence of significant cardiac or renal abnormalities. Intervention adherence was assessed through interviews and pharmacy records, while medication tolerability was monitored for adverse effects. Adverse events included transient hypotension episodes and mild medication side effects, which were managed appropriately. The patient’s response to therapeutic interventions and subsequent outcomes reflected effective management of the hypertensive crisis and underlying kidney disease.

## 8. Discussion

This case report presents valuable insights into managing a hypertensive crisis in a patient with underlying kidney disease, shedding light on strengths and limitations. A significant strength lies in its comprehensive assessment of the intricate relationship between hypertension and kidney dysfunction, emphasizing the critical importance of addressing both conditions concurrently for optimal outcomes. The detailed diagnostic assessment and therapeutic interventions outlined in the case contribute to the existing medical literature by offering a holistic view of clinical decision-making in such complex scenarios. However, limitations include the lack of long-term follow-up data and potential bias associated with retrospective case reporting. The scientific rationale for conclusions underscores the role of early intervention and tailored management strategies to prevent hypertensive crises and mitigate renal complications. This case serves as a reminder that successful outcomes necessitate a multidisciplinary approach that acknowledges the synergistic impact of hypertension and kidney disease.

### 8.1. Relevant medical literature

The findings of this case align with previous studies emphasizing the link between hypertensive crises and underlying kidney disease.^[1]^ Similar reports have highlighted the challenges of managing blood pressure surges while safeguarding renal function.^[2]^ Early recognition and prompt antihypertensive therapy have improved outcomes in hypertensive emergencies.^[3]^ The complexity of managing patients with comorbidities underscores the significance of interdisciplinary collaboration.^[2]^

### 8.2. Primary “Take-Away” lessons

This case report underscores the necessity of a comprehensive approach to managing hypertensive crises in patients with underlying kidney disease. It emphasizes the crucial roles of early recognition, tailored interventions, and close monitoring to prevent further complications and ensure favorable outcomes. Integrating knowledge from multiple specialties and continuous patient education is pivotal in successfully navigating the intricate interplay between hypertension and renal dysfunction.

## 9. Patient perspective

Upon my arrival at the emergency department, I was overwhelmed by the severity of my symptoms—the pounding headache, blurry vision, and chest discomfort. The medical team swiftly took action, and I was immediately administered medications through an IV. The relief was almost palpable as the intense pressure in my head began to subside, and I felt a sense of reassurance, knowing that steps were being taken to address the crisis. As the days went by, the healthcare providers closely monitored my condition, explaining each adjustment in medication and assuring me that my blood pressure was gradually coming under control. Despite some moments of dizziness and concern, I appreciated their constant attention and explanations, which helped alleviate my anxiety. Through the process, I gained a deeper understanding of the importance of medication adherence, lifestyle modifications, and regular check-ins with my healthcare team. While it was a challenging experience, I am grateful for the expertise and care that guided me back to a path of stability and better health.

## 10. Informed consent

The patient provided informed consent for their medical treatment and participation in this case report. The patient was thoroughly informed about the purpose of the case report, its potential implications, and the utilization of their de-identified medical information for educational and research purposes. The patient’s questions and concerns were addressed, and they were given ample time to make an informed decision. The patient understood that their participation was voluntary and that they could withdraw consent at any time without affecting their medical care. Consent was obtained in accordance with established ethical guidelines and documented appropriately.

## Author contributions

**Conceptualization:** Chukwuka Elendu, Dependable C. Amaechi, Tochi C. Elendu, Yuliana Paola Oros Sucari, Jennifer O. Ibhiedu.

**Data curation:** Chukwuka Elendu, Dependable C. Amaechi, Tochi C. Elendu.

**Formal analysis:** Chukwuka Elendu, Dependable C. Amaechi, Tochi C. Elendu.

**Funding acquisition:** Kanishk Dang.

**Investigation:** Chukwuka Elendu, Dependable C. Amaechi, Tochi C. Elendu, Kanishk Dang.

**Methodology:** Chukwuka Elendu, Dependable C. Amaechi, Tochi C. Elendu.

**Project administration:** Chukwuka Elendu, Dependable C. Amaechi, Tochi C. Elendu, Sehajmeet Kaur Saggi, Kanishk Dang.

**Resources:** Chukwuka Elendu, Dependable C. Amaechi, Tochi C. Elendu, Kanishk Dang.

**Software:** Chukwuka Elendu, Dependable C. Amaechi, Tochi C. Elendu, Kanishk Dang.

**Supervision:** Chukwuka Elendu, Dependable C. Amaechi, Tochi C. Elendu, Yuliana Paola Oros Sucari, Sehajmeet Kaur Saggi, Kanishk Dang, Jennifer O. Ibhiedu.

**Validation:** Chukwuka Elendu, Dependable C. Amaechi, Tochi C. Elendu, Kanishk Dang, Jennifer O. Ibhiedu.

**Visualization:** Chukwuka Elendu, Dependable C. Amaechi, Tochi C. Elendu, Yuliana Paola Oros Sucari, Sehajmeet Kaur Saggi, Kanishk Dang, Jennifer O. Ibhiedu.

**Writing – original draft:** Chukwuka Elendu, Dependable C. Amaechi, Tochi C. Elendu, Kanishk Dang.

**Writing – review & editing:** Chukwuka Elendu, Dependable C. Amaechi, Tochi C. Elendu, Yuliana Paola Oros Sucari, Sehajmeet Kaur Saggi, Kanishk Dang, Jennifer O. Ibhiedu.
